# Protein targets in red complex pathogens for catechin

**DOI:** 10.6026/973206300171105

**Published:** 2021-12-31

**Authors:** T Cibikkarthik, AS Smiline Girija, J Vijayashree Priyadharsini

**Affiliations:** 1 Saveetha Dental College, Saveetha Institute of Medical and Technical Sciences (SIMATS), Saveetha University, Chennai, India

**Keywords:** Catechin, red complex pathogens, multi-drug transporters, efflux pumps

## Abstract

The development of antimicrobial drug resistance has encouraged scientists to develop alternate methods to combat infectious pathogens associated with dental diseases. Therefore, it is of interest to predict interactions for catechin (a plant derived compound) with protein targets in the red complex pathogens using computer aided network tools. However, in vitro and in vivo studies are warranted to confirm the antimicrobial effect of catechin (gallocatechin, epicatechin, epigallactocatechin (EGC) and gallolyl catechins) on the dental pathogens.

## Background:

Drug resistance to pathogens has reached alarming numbers in recent times. A global burden of antibiotic resistant organisms in the community as well as hospital settings is seen due to indiscriminate use of antibiotics. Oral micro flora is a complex environment wherein there is interplay between host and pathogens causing infectious diseases. Several pathogens of the oral cavity such as Enterococcus faecalis, Streptococcus mutans etc. are known to contain proteins responsible for drug resistant phenotypes [1,2]. Selective pressure rendered by the antibiotic laden environment is mainly responsible for the emergence and resurgence of such resistant forms. Traditional or folk medicines have been used since decades to treat mild infections and disorders. The advantages of traditional medicine include (a) phyto compounds are derived from plants; (b) non-toxic compared to synthetic drugs, (c) metabolized by the biological system with antimicrobial activity against pathogens. The red complex pathogens are a group of microorganisms mostly associated with severe periodontal infections. These organisms are usually found along with other bacteria in the periodontal pockets and cause destruction of periodontal tissues in a synergistic way [3]. Mouthwashes incorporated with bioactive principles from plants can be used as a substitute so as to break the communication between these pathogens. Several plant-derived compounds have been tested against various pathogens [4]. The biological properties of green tea have catechin including gallocatechin, epicatechin, epigallactocatechin (EGC), gallolyl catechins etc. EGC is the most abundant form and represents about 50% of all known types [5]. Bai et al. (2016) [8] showed the antimicrobial effect of epigallacto catechin (EGCG) on canine oral bacteria. EGCG was found to possess a promising growth and biofilm inhibiting property [6]. Several protein components of bacteria interacting with bioactive and synthetic compounds using computer-aided tools have been known [7, 8]. Therefore, it is of interest to find the molecular targets of catechin on red complex pathogens.

## Materials & Methods:

The mechanism underlying the anti-microbial activity of catechin against red complex pathogens is investigated using available computer aided predictive tools.

## Strains used in the study:

**Figure 1:**
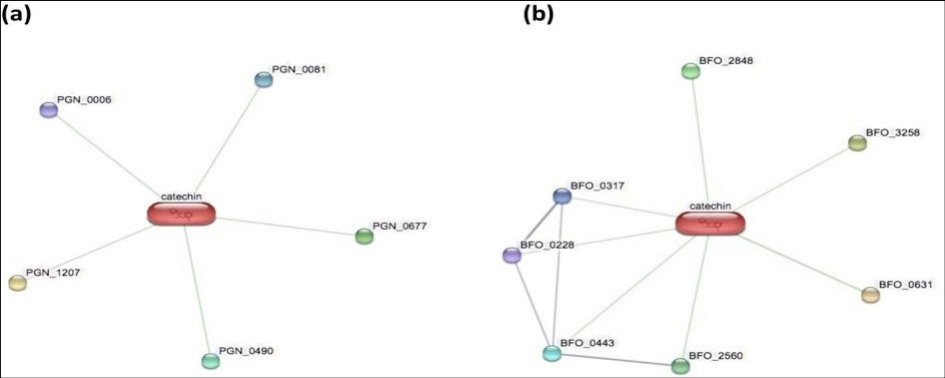
Interaction of catechin with the protein repertoire of red complex pathogens such as (a) Porphyromonas gingivalis and (b) Tannerella forsythia

The strains of red complex pathogens sich as Porphyromonas gingivalis ATCC 33277, Treponema denticola ATCC 35405 and Tannerella forsythia ATCC 43037 available in the STITCH database were selected for the analysis (Figure 1, Table 1 - see PDF).

## Analyzing protein interaction network:

The interactions between catechin and the protein repertoire of Streptococcus mutans UA159 and Enterococcus faecalis V583 was used for predicting the functional class and virulent nature of the proteins [9]. The FASTA format of protein sequences was retrieved from the National Centre for Biotechnology Information (NCBI).

## Prediction of functional class for interacting proteins:

VICMpred classifies the microbial proteins into four major classes such as (1) virulence factors; (2) information and storage processing; (3) cellular process and (4) metabolism. The virulence factors are identified using a support vector machine (SVM) algorithm, which classifies proteins based on their amino acid composition and sequence pattern [10].

## Prediction of virulence properties of interacting protein:

VirulentPred is a yet another SVM based method, used for automated prediction of virulent proteins based on the sequences [11]. The scores with positive predicted values are more often categorized as virulent protein and those with negative predicted values are categorized as avirulent proteins.

## Results and Discussion:

Catechin is a major component of green tea known for its antioxidant, anti-inflammatory, anti-proliferative activities. Bai et al. has shown the antimicrobial activity of catechin against a canine dental pathogen [8]. It is of interest to identify the possible molecular mechanisms underlying the antimicrobial effect of catechin using computational tools. The phytocompound catechin was found to target multiple proteins of Porphyromonas gingivalis and Tannerella forsythia. Data shows that catechin did not have interaction with the protein pool of Treponema denticola. However, the multidrug transporter MatE of T. forsythia was identified as a virulence factor using VICMPred tool and found to be avirulent by VirulentPred analysis. The MatE protein belongs to the multidrug efflux family protein. It is a transmembrane protein, which enables active transport of solutes by a mechanism. EGCG, a form of catechin has inhibitory activity on infections caused the multi-drug resistant Staphylococcus aureus [12,13]. Falcinelli et al. showed the bactericidal effect of EGCG against both the attenuated B. anthracis and the virulent encapsulated B. anthracis Ames strain [14]. Maria et al. showed significant antimicrobial activity of EGCG against both gram negative (Escherichia coli) and gram positive (Staphylococcus aureus) [15]. The synergistic activity of catechin hydrate in combination with different antibiotics was tested against the clinical strains of Staphylococcus aureus is known. The results show that catechin hydrate produced significant antimicrobial activity in combination with clindamycin and erythromycin. These data shows the antibacterial effect of catechin on human pathogens.

## Conclusion:

We document the predicted interaction targets for catechin (a plant derived compound) with protein targets in the red complex pathogens using computer aided network tools. However, in vitro and in vivo studies are necessary to verify the antimicrobial effect of catechin (gallocatechin, epicatechin, epigallactocatechin (EGC) and gallolyl catechins) on the dental pathogens.

## References

[1] Vijayashree Priyadharsini J (2018). Heliyon.

[2] Priyadharsini JV (2018). Arch Oral Biol.

[3] Suzuki N (2013). Int J Dent.

[4] Pedrazzi V (2015). Scientific World Journal.

[5] Zou C (2021). Food Chem.

[6] Ushanthika T (2021). Nat Prod Res.

[7] Vijayashree PJ (2019). J Periodontol.

[8] Bai L (2016). J Vet Med Sci.

[9] Szklarczyk D (2016). Nucl Acid Res.

[10] Saha S (2006). Gen Prot Bioinform.

[11] Garg A (2008). BMC Bioinform.

[12] Stapleton PD (2004). Int J Antimicrob Agents.

[13] Nance CL (2003). Aller Clin Immunol.

[14] Falcinelli SD (2017). FEMS Microbiol Lett.

[15] Miklasinska M (2016). Molecules.

[16] Wu M (2021). Pathogens.

